# Evaluation of cortical and trabecular bone structure of the mandible in patients with ankylosing spondylitis

**DOI:** 10.1038/s41598-023-47233-2

**Published:** 2023-11-13

**Authors:** Melike Gulec, Mediha Erturk, Melek Tassoker, Muserref Basdemirci

**Affiliations:** 1 Department of Dentomaxillofacial Radiology, Faculty of Dentistry, Karamanoğlu Mehmetbey University, Karaman, Turkey; 2 Department of Dentomaxillofacial Radiology, Faculty of Dentistry, Necmettin Erbakan University, Konya, Turkey; 3 Department of Medical Genetics, Konya City Hospital, Konya, Turkey

**Keywords:** Anatomy, Medical research, Signs and symptoms

## Abstract

This study aimed to examine the difference between the fractal dimension (FD) values of the mandibular trabecular bone and the panoramic mandibular index (PMI), mandibular cortical index (MCI) and mandibular cortical thickness (MCW) of patients with ankylosing spondylitis (AS) and healthy control group. A total of 184 individuals (92 cases, 92 controls), were examined in our study. PMI, MCI, and MCW values were calculated on panoramic images of all individuals. For FD values, the region of interest (ROI) was selected with the size of 100 × 100 pixels from the right-left gonial and interdental regions and 50 × 50 pixels from the condylar region. Degenerative changes in the temporomandibular joint (TMJ) region were recorded. PMI, MCI, and MCW values showed statistically significant differences between the groups (p = 0.000, p < 0.001). The radiological signs of mandibular cortical resorption were more severe in the case group than in the control group. PMI and MCW values were found to be lower in the case group than in the control group. It was determined that the number of C3 and C2 values, among the MCI values, was higher in the case group. Only the FD values of the ROI selected from the condyle region were found to be statistically significant and were lower in the case group (p = 0.026, p < 0.05). Degenerative changes in the TMJ region were significantly more frequent in the case groups (p = 0.000, p < 0.001). The fact that the mandibular cortex shows more resorptive features in individuals with AS may require further evaluation in terms of osteoporosis. Because of the low FD values of the condylar regions of these patients and the more frequent degenerative changes, the TMJ region should be followed carefully. Detailed examination of the mandibular cortex and condylar region is beneficial in patients with AS for screening and following osteoporotic changes in these individuals, which is essential for the patient’s life quality.

## Introduction

Ankylosing spondylitis (AS), the etiology of which is unclear, is a chronic rheumatic disease characterized by pathological bone remodeling and inflammation^1^. AS affects sacroiliac joints in its early stages and axial skeleton and peripheral joints in the later stages of disease development. AS often causes inflammation in the entheses, which is defined as the area where tendons and ligaments attach to the bone. AS is prevalent in younger men and is a systemic disease that can manifest itself not only with musculoskeletal involvement, but also with organ involvement from different systems, such as the eye, heart, intestine, and lung. AS reduces quality of life as it causes a significant loss of function^2^.

Osteoporosis caused by ankylosis, sclerotic healings, and local trabecular bone erosions can develop in the affected bone by disruptin the balance of construction and destruction within the scope of the pathological bone remodeling process observed in AS^3,4^. It has been considered paradoxical that a disease that manifests itself with cortical bone formation that might cause ankylosis is also associated with bone loss. The inflammation observed in AS causes trabecular bone loss and leads to osteopenia and osteoporosis. Osteoporosis can be explained by the effect of inflammation on the bone remodeling cycle^5^. Osteopenia and osteoporosis due to decreased bone mineral density (BMD) are considered to be the very common manifestations of AS^5^. Studies have reported that the prevalence of osteoporosis associated with AS ranges from 18 to 90%^6,7^. Osteoporosis associated with AS is extremely important in terms of causing fractures and deformities (especially in the axial skeleton) and increasing morbidity and mortality rates^8^.

As an alternative to BMD measurements, such as dual-energy X-ray absorptiometry (DXA) and quantitative computed tomography (QCT), which are considered the gold standard in the diagnosis of osteoporosis, but are difficult to access, invasive, time-consuming, and expensive^9^, mandibular radiomorphometric indices are viable substitute. Indices including mandibular cortical index (MCI), mandibular cortical width (MCW), and panoramic mandibular index (PMI) are used to detect osteoporosis-induced changes in cortical bone. In previous studies, it has been reported that the MCI value is associated with mandibular porosity and can be used as a screening method for osteoporosis^9–11^. In the literature, some studies associate a decrease in MCW value with a decrease in skeletal BMD^12,13^. PMI is the ratio of the mandibular cortex thickness to the distance between the mental foremen and the inferior mandibular cortex^14^.

Complex structures that cannot be defined by standard geometric shapes, such as squares, rectangles, and triangles in Euclidean geometry and that exhibit similar properties when viewed from different scales are called fractals^15^. The fractal analysis makes it possible to obtain a quantitative degree of complexity, expressed as fractal dimension (FD), by analyzing self-repeating patterns within a given structure. There are many studies evaluating the changes in the micro-architecture of trabecular bone with fractal analysis^15–18^. As a result of local erosions seen in osteoporosis cases, it is expected that the degree of complexity of the internal structure will decrease due to the decrease in micro-branching in the trabecular bone and the increase in porosity, thus, the FD value will decrease^5^.

Early detection of osteoporotic changes in patients with AS is important in terms of patient prognosis. Although there are several studies on the osteoporotic effects in axial skeletal bones in the literature^5,19,20^, there is a lack of data evaluating the mandibular cortical and trabecular structure in individuals with AS. This study aimed to compare mandibular trabecular bone FD values and mandibular radiomorphometric index values from panoramic radiographs of patients with AS versus healthy control subjects.

## Methods

### Sample selection and study design

This study included 184 individuals (aged 19–54 years), 92 AS patients and 92 control individuals, whose panoramic radiography was obtained for dental diagnosis and treatment procedures between 2014 and 2023. Panoramic radiographs obtained during the routine radiographic examination of individuals who applied to the Department of Dentomaxillofacial Radiology of Necmettin Erbakan University Faculty of Dentistry for various dental reasons, were examined retrospectively. No additional X-ray exposure was applied to the patients for this retrospective study. Anamnesis records in the database were used to form the case and control groups. Patients using glucocorticoids or tumor necrosis factor-α (TNF-α) inhibitors were excluded from the study. The control group included systemically healthy individuals who matched the case group in terms of age and gender. This study was approved by the human subjects ethics board of Necmettin Erbakan University Faculty of Dentistry, Pharmaceuticals, and Non-Medical Devices Ethics Committee (2023/297) and was conducted in accordance with the Helsinki Declaration of 1975, as revised in 2013. The informed consent was waived due to retrospective nature of the study by the Necmettin Erbakan University Faculty of Dentistry, Pharmaceuticals, and Non-Medical Devices Ethics Committee.

In addition to FD analysis of the trabecular bone, MCI, MCW, PMI, and temporomandibular joint (TMJ) surface changes were analyzed in the study participants.

### Radiomorphometric indices (MCI, MCW, and PMI)

According to the MCI, the portion of the mandibular cortex distal to the mental foramen was evaluated on three different scores according to the following criteria^21^. In MCI, panoramic radiographs are evaluated and graded separately for right and left, and then a single grade was assigned for each panoramic radiograph. In determining the final class, the class with more morphologic destruction was preferred to the class with less destruction.

**Figure 1:**
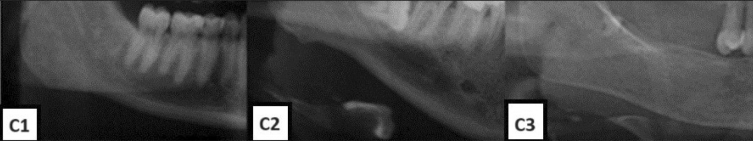
MCI values of 3 different cropped panoramic images are C1, C2, and C3 from right to left, respectively. C1: the right mandibular endosteal margin of the cortex is normal. C2: stratification is observed in the cortex of the right mandibular endosteal margin. C3: the endosteal margin is markedly porous.

C1: the endosteal margin of the cortex is smooth. C2: unilaterally and bilaterally, there are resorption cavities and stratification (1–3) at the endosteal margin. C3: the endosteal margin is markedly porous (Fig. 1).

**Figure 2:**
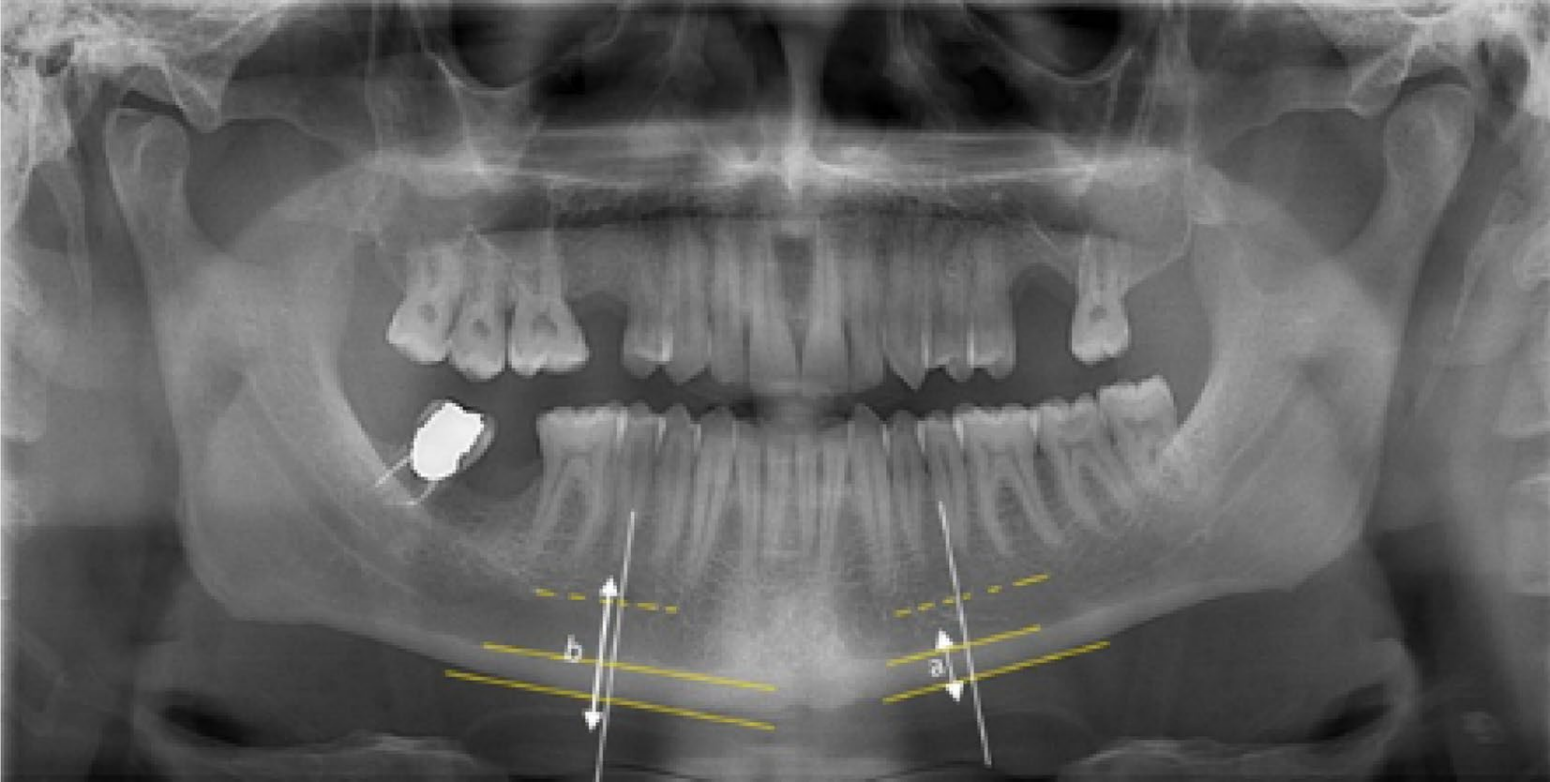
MCW is the width indicated by ‘a’ and the ‘a/b’ ratio gives the PMI result.

For MCW measurement, a line was drawn tangent to the mandibular cortex at the level of the mental foramen, in the premolar region. A second line was drawn through the foramen mentale and perpendicular to this line. The distance from the intersection of these two lines to the lower border of the mandible (a) was used for MCW^22^. Two different values, right and left, were recorded (Fig. 2).

For the PMI calculation, the distance between the inferior border of the foramen mentalis and the inferior border of the mandible (b) was measured. PMI was calculated by dividing the cortical thickness of the mandible (a) by the distance from the inferior border of the mental foramen to the inferior border of the mandible (a/b) (Fig. 2)^14^. PMI was measured separately in the right and left mandible and the mean value was calculated and recorded as a single value for each participant^23^.

### Radiographic image processing: fractal analysis

For the standardization of the radiographs, the dimensions of all images were adjusted to 2836 × 1500 pixels using Adobe Photoshop CS5^24^ (Adobe Systems Inc., San Jose, CA). For fractal analysis, ImageJ v1.41, a version of the National Institutes of Health Image software with 64-bit Java for Windows, was used (NIH, Bethesda, MD, USA, can be downloaded at https://imagej.nih.gov/ij/download.html)^25^. The measurements were repeated twice at 15-day intervals by the same oral radiologist (MG) who has 8 years of oral radiology experience.

**Figure 3:**
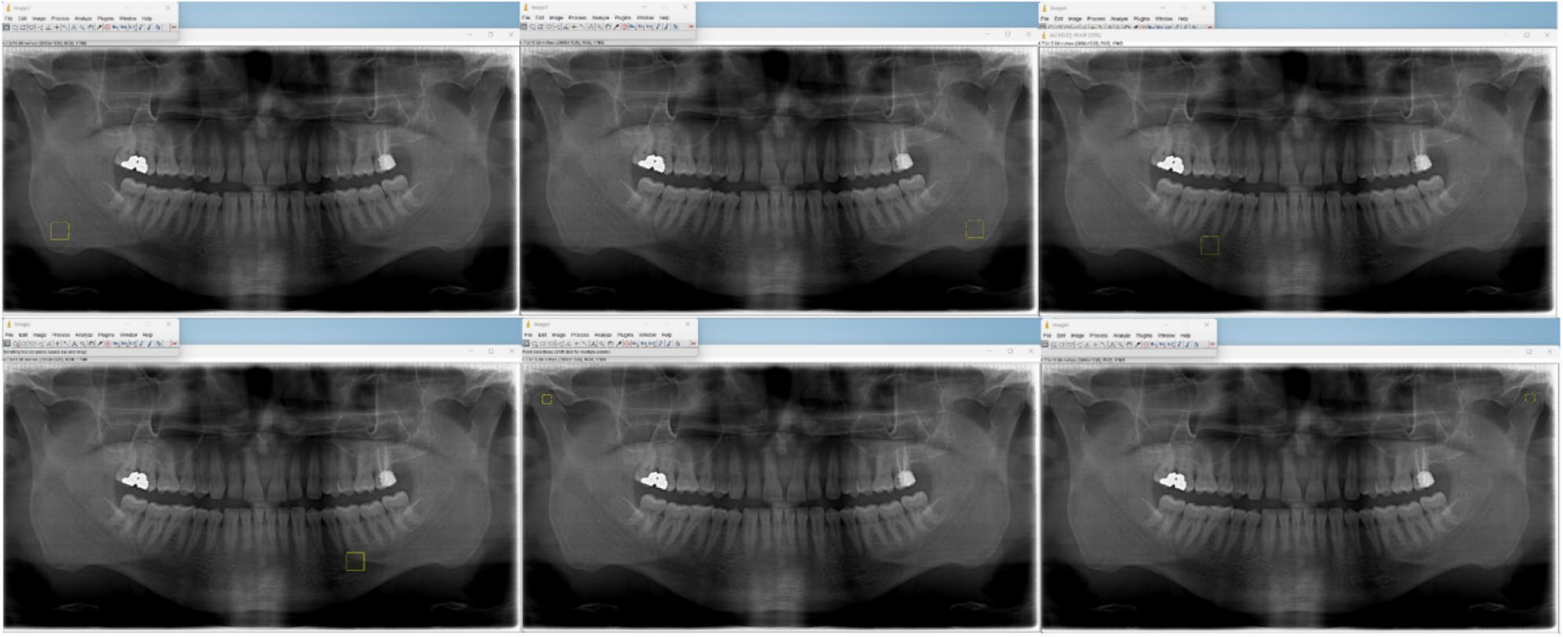
Identified ROIs are marked on the ImageJ program.

On panoramic radiographs:

1. 100 × 100 pixels from the right and left mandibular angulus.
2. 100 × 100 pixels from the area between the apical regions of the right-left mandibular second premolars and first molars (excluding the periodontium of the teeth and the cortical borders of the mandibular canal).
3. A total of six regions of interest (ROIs) of 50 × 50 pixels were selected from the right and left condylar regions (Fig. 3).

**Figure 4:**
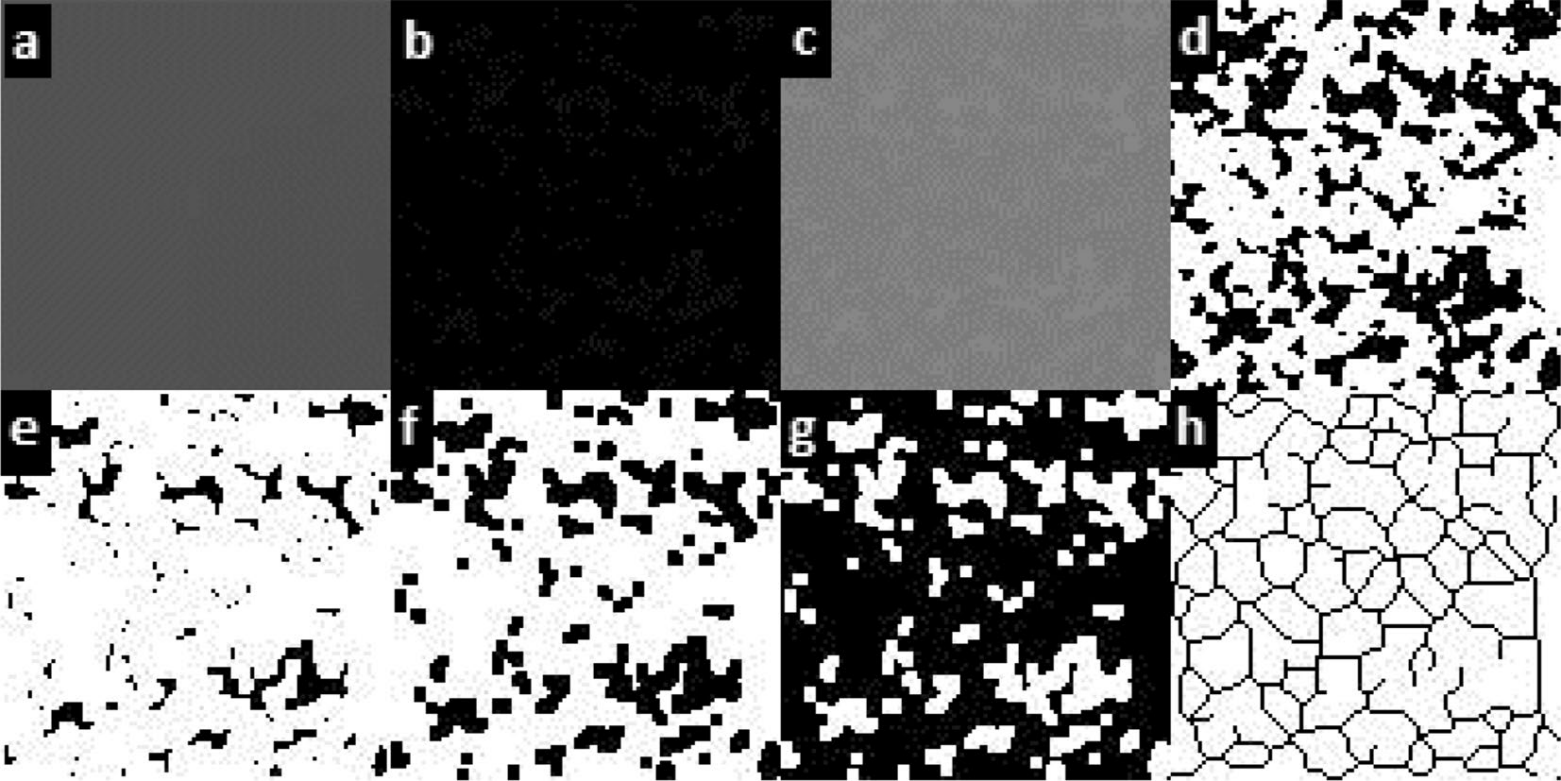
(**a**) Blurring. (**b**) Subtracting the blurred image from the original image. (**c**) Adding 128 shades of gray. (**d**) Converting to black and white image. (**e**) Reducing noise with Erode. (**f**) Expanding with Dilate. (**g**) Inverting colors. (**h**) Converting to skeletal format.

The box-counting method described by White and Rudolph^26^ was used to calculate the FD values (Fig. 4).

### TMJ examinations

**Figure 5:**
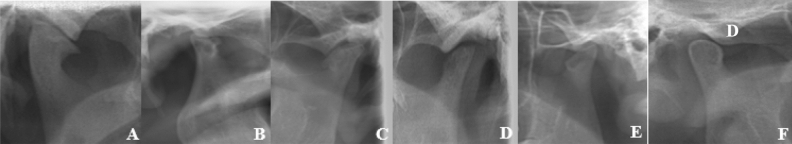
(**A**) osteophyte formation. (**B**) subchondral cyst. (**C**) sclerosis. (**D**) erosion. (**E**) surface flattening. (**F**) healthy TMJ condyle area.

Degenerative changes in the condyle were analyzed under six headings: osteophyte formation, subchondral cyst development, subchondral sclerosis, surface erosion, flattening of the condyle, and healthy (Fig. 5)^27^. The right and left condyles were evaluated separately and if the pathology was observed in any condyle, the overall score was indicated as ‘surface change present’.

### Imaging and calibration

Panoramic radiographs were obtained with 2D Veraviewpocs (J MORITA MFG corp, Kyoto, Japan) according to the irradiation parameters of 70 kV, 5 mA, and 15 s within the routine protocol established following the manufacturer's recommendations. For calibration, the patient's Frankfurt plane was adjusted to be parallel to the floor and the patient's sagittal line was adjusted to be perpendicular to the floor. This panoramic unit has a magnification factor of 1.3; therefore, the MCW and PMI measurements were calibrated to 1.3. Images with poor image quality due to positioning errors were not included in the study. Turcasoft Software @2021 Dental Information Management System (Turcasoft Software, Samsun, Turkey) was used for MCW and PMI measurements.

### Statistical analysis

SPSS v.21 (IBM Corp., Armonk, NY, USA) was used for data analysis. Descriptive statistics (mean, minimum, maximum, and standard deviation) were calculated and the Chi-squared test was used to analyze categorical data (MCI observations) between case and control groups. The Shapiro–Wilk test was used to determine the normality. Normality was violated and non-parametric Mann–Whitney U test was used to compare the measurements (MCW, PMI, and FD) between the case and control groups. The measurements were repeated twice at 15-day intervals by the same oral radiologist (MG). Intraobserver agreement was determined using 25% of the patients. Intraclass correlation coefficient (ICC) was applied to test intraobserver agreement. The significance of the test results was determined according to p < 0.05.

### Ethics approval

This study was conducted at the Faculty of Dentistry, Necmettin Erbakan University, Department of Dentomaxillofacial Radiology, with the approval of the Ethics Committee (No. 2023/297) and was performed according to the stipulations laid out by the Declaration of Helsinki.

## Results

A total of 184 individuals (68 males and 116 females) were examined in the study and separated into case (92) and control (92) groups, matched by age and gender. The mean age was 39.8 ± 8.6 years.

MCW, PMI, and MCI values related to the evaluation of the mandibular cortex showed statistically significant differences between the case and control groups (p = 0.000, p < 0.001; Tables 1 and 2). The radiological signs of mandibular cortical resorption were more severe in the case group than in the control group. PMI and MCW values were found to be lower in the case group than in the control group (Table 1). It was also determined that the C3 and C2 values, among the MCI values, was higher in the case group (Table 2).

**Table 1:** PMI and MCW values in case and control groups.

	Patients	N	Mean ± SD	Mann–Whitney U test
PMI	Control	92	0.293 ± 0.05	p = 0.000*
	Case	92	0.242 ± 0.06	
	Total	184		
MCW	Control	92	3.390 ± 0.63	p = 0.000*
	Case	92	3.040 ± 0.91	
	Total	184		

PMI, panoramic mandibular index; MCW, mandibular cortical width; SD, standard deviation.

*p value is significant at p < 0.001.

**Table 2:** The distribution of MCI scores in case and control groups.

		MCI			Total	χ2
		C1	C2	C3		
Patients	Control	36	48	8	92	
	Case	16	55	21	92	p = 0.000*
Total		52	103	29	184	

MCI, mandibular cortical index.

*p value is significant at p < 0.001.

The mean FD values measured from the right and left sides of the patients are given in Table 3. While the FD values of the trabecular bone did not show a statistically significant difference between the case–control group in the ROIs selected from the gonial and interdental regions, the FD values of the ROI selected from the condyle region were lower in the case group (mean: 1.38 ± 0.06) than in the control group (mean: 1.41 ± 0.07; p = 0.026, p < 0.05). The intraobserver agreement was good (ICC = 0.76).

**Table 3:** FD values for the case and control group.

	Patients	N	Mean FD ± SD	Mann–Whitney U test
Gonial	Control	92	1.43 ± 0.05	p = 0.485
	Case	92	1.44 ± 0.05	
	Total	184		
Interdental	Control	92	1.39 ± 0.04	p = 0.966
	Case	92	1.38 ± 0.05	
	Total	184		
Condyle	Control	92	1.41 ± 0.07	p = 0.026*
	Case	92	1.38 ± 0.06	
	Total	184		

FD, fractal dimension; SD, standard deviation.

*p-value is significant at p < 0.05.

Degenerative changes in the TMJ region (osteophytes, subchondral cysts, sclerosis, erosion, and flattening) were significantly more frequent in the case groups (p = 0.000, p < 0.001). The frequency of degenerative changes was 26.1% (24/92) in the control group and 68.5% (63/92) in the case group. The distribution of degenerative changes is given in Table 4. The most common TMJ degeneration was flattening.

**Table 4:** The distribution of TMJ degenerations.

Type of degeneration		Right TMJ		Total	Left TMJ		Total
		Control	Case		Control	Case	
	No degeneration	77	50	127	77	41	118
	Osteophytes	7	13	20	4	7	11
	Subsclerosis	0	1	1	2	0	2
	Subchondral cyst	0	3	3	1	4	5
	Erosion	0	4	4	0	3	3
	Flattening	8	21	29	8	37	45
Total		92	92	184	92	92	184

## Discussion

In this retrospective study, it was determined that PMI and MCW values in the case group were significantly lower than in the control group, and C2 and C3 MCI values (which are associated with resorptive changes), were more frequent in the case group. Additionally, FD values measured from the condylar region were significantly lower in the case group and degenerative changes in the TMJ region were significantly more frequent in the case group.

AS is a chronic, progressive, inflammatory disease that usually affects young adults (especially in the second and third decades)^28^. AS is also more common in men and is the most common of the prototypical seronegative spondyloarthropathies with a prevalence of 0.9%^28^.

Osteoporosis associated with AS has a complex and multifactorial etiology^2^. These mechanisms include mechanical, biochemical, hormonal, and genetic factors. One of the most accepted theories is the effect of chemical inflammatory mediators. In this disease, it has been reported that there is a strong release of cytokines produced by macrophages, such as TNF-α and interleukin-6 (IL-6), are associated with synovial inflammation and joint damage and affect osteoblasts and osteoclasts in all bone tissue^29^. In case–control studies, serum levels of IL-6 and TNF-α were found to be increased in patients with AS^30^. As for genetic factors, the HLA-B27 gene was found to be positive in 80–90% of individuals with AS. Studies have reported that the risk of AS in first-degree relatives is more than 52 times higher than in independent cases^31^. Other factors blamed in the etiology are decreased mobility, use of corticosteroids, disorders in calcium and vitamin D metabolism, deficiency of sex steroids, and silent bowel disease^1,7,32^.

Osteoporosis is a common complication in AS patients^5^. Bone strength depends on trabecular and cortical microarchitecture and also, bone mineral content. Osteoporosis can negatively affect both of these factors, leading to loss of total bone mass and disruption of trabecular microarchitecture and specific dissolution effects on osseous tissues (such as thinner trabeculae and higher trabecular separation) and reduced cortical thickness^33^. The resulting osteoporotic fractures significantly affect the quality of life in these patients^34,35^.

When studies on AS and osteoporosis are examined, it is seen that they are generally conducted on the axial skeleton. However, recent studies have revealed the relationship between axial and peripheral bone loss and reported that AS-induced osteoporosis causes peripheral fractures in addition to increasing vertebral fractures^33,36^. Klingberg et al.^33^ reported that AS-induced pathological bone formation is localized, but AS-induced trabecular bone loss is general, which involves both central and peripheral bones. In addition to trabecular thinning, perforation, and trabecular loss, an increase in cortical porosity is observed in the osteoporotic process. In addition to BMD measurements, understanding the trabecular microarchitecture is vital in the diagnosis of osteoporosis^34,35^. Researchers have stated that the mineral density in the jaw bones is parallel to the mineral density in other bones of the body^37^. Therefore, in our study, the mandibular cortical and trabecular bone structure was analyzed in individuals with AS.

Currently used BMD measurements include DXA, QCT, and film densitometry^38^. Among these, DXA is considered the gold standard for the diagnosis and follow-up of osteoporosis because it can measure both trabecular and cortical BMD and determine the risk of osteoporotic fracture^39^. Despite all these advantages, the cost and lack of accessibility for all segments of the population limits the use of DXA^40^. Drozdzowska et al.^41^ reported that the mandible was more affected by osteoporosis than other skeletal bones in a longitudinal study of postmenopausal women with a follow-up of 28 months. Hildebolt^42^ advocated the development of inexpensive and easily accessible methods for sensitive and specific assessment of oral bone loss. These findings, coupled with the widespread and non-invasive nature of dental radiologic applications (such as panoramic radiographs), are important for the opportunity to predict osteoporosis and osteopenia in large populations using accessible, lower radiation dose, and relatively inexpensive diagnostic and follow-up methods. For this purpose, this study investigated whether mandibular radiomorphometric indices and FD value calculated on panoramic radiographs can be used in the preliminary diagnosis and evaluation of osteoporosis.

Despite some limitations, such as the two-dimensional nature of the panoramic radiograph and the fact that image quality is affected by patient positioning mistakes and is partly dependent on the experience of the observer^43^, most studies in the literature have reported that mandibular radiomorphometric indices are also useful in the evaluation of osteoporosis^44–46^. In one study, the MCW value was found to be low in individuals with osteoporosis, whereas an increase in MCW value was interpreted as a decrease in the risk of osteoporosis^12,13^. In another study, a high MCI score that evaluates the posterior cortex of the mandible, was associated with low BMD^47^. Nemati et al.^48^ calculated PMI, MCW, and MCI values from panoramic radiographs of 90 postmenopausal women (30 osteoporotic, 30 osteopenic, and 30 controls) and measured BMD with DXA. They concluded that MCW, PMI, and MCI have a high diagnostic value in predicting low BMD and that a 1 mm reduction in MCW increases the probability of decreased BMD (osteoporosis and osteopenia) up to 3.22 times. In a study conducted by Kiswanjaya et al.^49^ in 2022, they measured BMD with quantitative ultrasound (QUS) and investigated whether PMI, MCW, and MCI values calculated from 371 panoramic radiographs could be used in osteoporosis risk prediction. As a result, they stated that PMI values less than 0.3, MCW values less than 3, and class 3 MCI values were associated with lower BMD. In this study, MCI, MCW, and PMI values were found to be statistically significantly different between the case and control groups. PMI and MCW values were found to be lower and MCI values were found to be higher in the case group compared with the control group.

FD analysis, which is increasingly used in medicine and dentistry because it is easily accessible and practical, offers the opportunity to detect deterioration in bone mass as an alternative to BMD measurements^9,50,51^. In this study, it was examined whether the FD values in the mandibular angulus, interdental, and condylar regions in AS patients differed from the control group. It was found that only the FD values calculated from the condyle region were significantly different between the case and control groups (p = 0.026, p < 0.05). It was observed that the FD values calculated from the case group (mean: 1.38 ± 0.06) were lower than the control group (mean: 1.41 ± 0.07). Although there are few studies in the literature suggesting that osteoporotic changes increase trabecular bone FD values^52,53^, it is thought that FD decreases in osteoporosis cases due to the increase in porosity and decrease in complexity as a result of dissolution in the trabecular structure^54–56^. Canger et al.^56^ applied fractal analysis to ROIs of 64 × 64 pixel images from condylar, gonial, and interdental regions in their study of 128 individuals (63 AS patients versus 65 controls) in 2023, and found that FD values calculated from gonial and condylar regions were significantly lower in the case group. As such, the FD values calculated from the condylar region in AS patients can be used in the onset and follow-up of osteoporotic changes in these individuals.

Studies have reported that TMJ diseases are more common in individuals with AS and the incidence of degenerative changes such as sclerosis and flattening increases when compared with healthy individuals^57,58^. Ramos-Remus et al.^57^ found that condylar erosion was associated with the severity and duration of AS and associated flattening and sclerosis with the remodeling process, which develops due to increased load on the TMJ. In our study, it was determined that degenerative findings were more common in the case group and the most frequently observed degenerative change was flattening. Careful examination of the TMJ region in long-term AS cases is very important for patient comfort.

In recent years, with the development of software, studies have been performed using advanced imaging methods such as CT^59^, CBCT^60^, and MRI^61^, where textural features are examined based on obtaining numerical data from digital images, and these inferences can be combined with clinical information. Nussi et al.^60^, examined the textural properties of the trabecular bone of the mandibular condyle region from CBCT images according to age and gender, and reported that there were significant textural differences according to gender, and that numerical values were lower in men. They attributed this to the fact that trabecular bone is more linear and thinner in women. In the study conducted by Ling et al.^62^ with CBCT, they stated that the trabecular bone regions selected from different ROIs of the jaws had different texture properties according to age and gender and that CBCT could be used in osteoporosis screening.

The main limitation of this study was the lack of DXA for analysis. Another limitation was retrospective design of the study. The lifestyle habits, body mass indexes, duration, and severity of the disease were not recorded. In clinical studies to be planned in this context, patients' physical activity levels, nutritional status, and disease period should be recorded. Furthermore, risk factors should be evaluated in detail. In future studies, it is recommended to investigate different image processing methods, such as texture analysis in addition to FD and cortical indices.

## Conclusion

Erosive changes were observed in the mandibular cortex and TMJ trabecular bone structure in patients with AS. The FD value measured from the trabecular bone of the TMJ region was lower in individuals with AS compared with healthy controls. Careful examination and radiologic follow-up of these structures are recommended for early detection and follow-up of osteoporosis in individuals with AS.

## Data Availability

The datasets generated and analyzed during the current study are available from the corresponding author on reasonable request.
